# *EXPANDER *– an integrative program suite for microarray data analysis

**DOI:** 10.1186/1471-2105-6-232

**Published:** 2005-09-21

**Authors:** Ron Shamir, Adi Maron-Katz, Amos Tanay, Chaim Linhart, Israel Steinfeld, Roded Sharan, Yosef Shiloh, Ran Elkon

**Affiliations:** 1 School of Computer Science, Sackler Faculty of Exact Sciences. Tel Aviv University, Tel Aviv 69978 Israel; 2 The David and Inez Myers Laboratory for Genetic Research, Department of Human Genetics, Sackler School of Medicine. Tel Aviv University, Tel Aviv 69978, Israel

## Abstract

**Background:**

Gene expression microarrays are a prominent experimental tool in functional genomics which has opened the opportunity for gaining global, systems-level understanding of transcriptional networks. Experiments that apply this technology typically generate overwhelming volumes of data, unprecedented in biological research. Therefore the task of mining meaningful biological knowledge out of the raw data is a major challenge in bioinformatics. Of special need are integrative packages that provide biologist users with advanced but yet easy to use, set of algorithms, together covering the whole range of steps in microarray data analysis.

**Results:**

Here we present the *EXPANDER *2.0 (EXPression ANalyzer and DisplayER) software package. *EXPANDER *2.0 is an integrative package for the analysis of gene expression data, designed as a 'one-stop shop' tool that implements various data analysis algorithms ranging from the initial steps of normalization and filtering, through clustering and biclustering, to high-level functional enrichment analysis that points to biological processes that are active in the examined conditions, and to promoter cis-regulatory elements analysis that elucidates transcription factors that control the observed transcriptional response. *EXPANDER *is available with pre-compiled functional Gene Ontology (GO) and promoter sequence-derived data files for yeast, worm, fly, rat, mouse and human, supporting high-level analysis applied to data obtained from these six organisms.

**Conclusion:**

*EXPANDER *integrated capabilities and its built-in support of multiple organisms make it a very powerful tool for analysis of microarray data. The package is freely available for academic users at

## Background

Gene expression microarrays are a prominent experimental tool in functional genomics. They have revolutionized biological research by providing genome-wide snapshots of transcriptional networks that are active in the cell. This opens the opportunity for gaining global, systems-level understanding of cellular processes. Microarray platforms for measuring the expression levels of most or all genes of an organism are available for a variety of organisms ranging from yeast to human. Experiments that use this technology typically generate overwhelming volumes of data, unprecedented in biological research, which makes the task of mining meaningful biological knowledge out of the raw data a major challenge. Hence, exploitation of gene expression data is fully dependent on the availability of advanced data analysis and statistical tools. Many algorithms and software tools for analysis of microarray data were developed in recent years, including sophisticated methods for signal extraction and array normalization [1,2], clustering [3,4], and statistical identification of over-represented functional categories [5] and promoter motifs [6,7]. At present, of special need are integrative software packages that provide users with a set of algorithms collectively covering the whole range of steps in microarray data analysis, thereby significantly boosting the analysis flow and the researcher's ability to deduce meaningful biological conclusions from the overwhelming volume of recorded data. Here we present the EXPANDER program suite for gene expression data analysis.

## Implementation

*EXPANDER *(EXPression ANalyzer and DisplayER), initially developed as a clustering tool [8], has been redesigned as a 'one-stop shop' tool for analysis of the data. *EXPANDER *2.0 integrates methods and algorithms that collectively cover different steps of the data analysis, ranging from the initial steps of normalization and filtering, through module detection by clustering and biclustering, to high-level analysis of functional enrichment and of promoter cis-regulatory elements. *EXPANDER *serves as the major platform in which we integrate various gene expression analysis algorithms that were developed in our lab, including CLICK for clustering [9], SAMBA for biclustering [10], PRIMA for promoter elements analysis [7], and TANGO for GO functional enrichment analysis (manuscript in preparation). In addition, EXPANDER implements various visualization utilities that accompany each of the analysis modules. Four basic design principles instructed us in the implementation of the package: First, the analysis flow should be highly streamlined. Second, although some of the modules are based on highly complicated algorithms, their use should be kept simple and results should be presented in an intuitive manner. Third, data analysis is expected to be done iteratively, allowing users to examine different parameter settings and clustering algorithms – therefore, special effort was put on efficient implementation of the algorithms. Forth, users should be freed from the burden of compiling annotation data required for the analysis. Therefore, EXPANDER not only implements the analysis algorithms, but also supplies users with all necessary annotation and sequence data.

**Figure 1:**
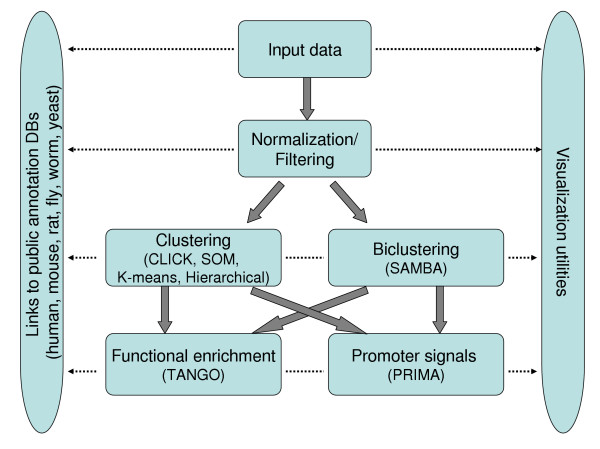
A high level summary of *EXPANDER*'s microarray data analysis flow and of the main algorithms implemented in each analysis step.

*EXPANDER *is available with genome-wide pre-processed functional Gene Ontology (GO) and promoter sequences data files for yeast, worm, fly, rat, mouse and human, supporting high-level analysis of data obtained from these organisms. *EXPANDER *supports analysis of both relative and absolute expression level datasets, the former generated by cDNA microarrays and the latter by, e.g., Affymetrix oligonucleotide arrays. The main utilities provided by *EXPANDER *and the major algorithms implemented in it are described in the Results section below. Figure 1 gives a high level summary of *EXPANDER*'s analysis flow and of the main algorithms implemented in each analysis step.

*EXPANDER *is implemented in Java. Most of the algorithms it runs were implemented in C. EXPANDER versions for Windows and UNIX are freely available for academic users.

## Results

In this section we describe the main analysis modules implemented in *EXPANDER*, and present a case analysis that demonstrates the strength of this package in deriving biological conclusions out of massive gene expression datasets.

### Normalization

The goal of this pre-processing step is the removal of technical biases among the analyzed chips. Currently, the default normalization scheme applied by Affymetrix software is the global scaling, which multiplies all intensities measured in a chip by a constant factor to bring the average/median intensity level in each chip to a predefined fixed level. However, several studies pointed out that global scaling is too naïve in many cases, and that more sophisticated normalization procedures accounting, e.g., for intensity-dependent bias, are required [11,12]. We implemented in *EXPANDER *two such methods: non-linear regression and quantiles equalization as described in [1]. Normalization of cDNA arrays requires intensity levels measured in both red and green channels. EXPANDER expects log ratios (Red/Green) as input when analyzing dual channels data. Therefore, normalization schemes in *EXPANDER *are available at this stage to one-channel datasets. Several novel normalization schemes are not yet implemented in *EXPANDER *(e.g., Variance Stabilizing Normalization (VSN) [13], Li-Wong invariant set normalization [14]). Users can load *EXPANDER *with data that were normalized using external tools.

### Filtering utilities

*EXPANDER *provides several commonly-used filtering options based on fold-change factors, minimal variation criteria, or choosing the n most variant genes, allowing the user to focus downstream analysis on the set of genes that show sufficient variation across the measured conditions.

### Cluster analysis

**Figure 2:**
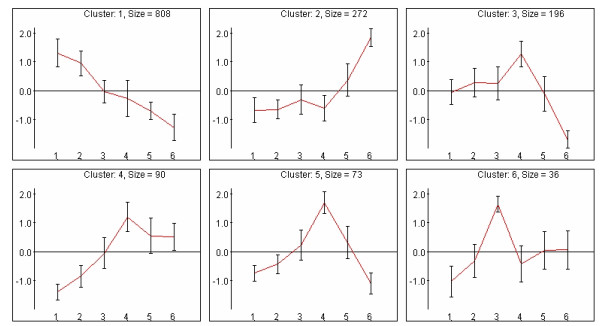
All-patterns display of a clustering solution. Each graph represents a specific cluster. The X-axis represents the measured conditions. The Y-axis represents (standardized) expression levels. Each cluster is represented by the mean expression pattern over all the genes assigned to it. Error bars denote ± 1 standard deviation. Clicking within a cell opens a window that lists the genes that are assigned to the cluster.

**Figure 3:**
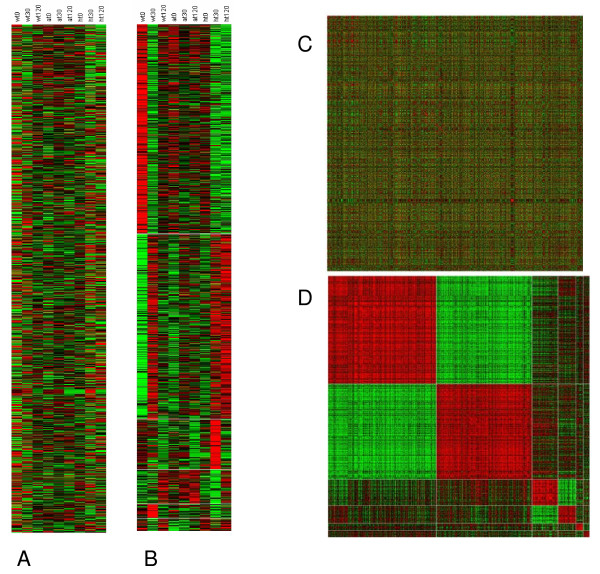
Matrix displays. (A) Unclustered expression matrix display. Each row corresponds to a gene, and each column to a biological sample. The color of the (i, j) cell in the matrix indicates the expression level of the ith gene in the jth sample. Green represents below-average expression level; Red represents above-average expression level (color scheme can be adjusted by the user). (B) The same dataset as in A, with genes ordered according to a clustering solution. Horizontal white lines separate the different clusters. (C) Unclustered similarity matrix display. The color of the (i, j) cell in the matrix represents the similarity between the expression patterns of the ith and the jth genes over all the samples (hence the matrix is symmetric). Red represents high similarity, and green represents low similarity. (D) Same as in C, with genes ordered on both axes according to a clustering solution. Clusters appear as distinct red blocks along the matrix diagonal, and similar clusters are manifested by off-diagonal reddish blocks.

Clustering algorithms applied to gene expression data partition the genes into distinct groups according to their expression patterns over the probed biological conditions. Such partition should assign genes with similar expression patterns to the same cluster (keeping the *homogeneity *merit of the clustering solution) while retaining the distinct expression pattern of each cluster (ensuring the *separation *merit of the solution). Cluster analysis eases the interpretation of the data by reducing its complexity and revealing the major patterns that underlie it. *EXPANDER *implements a few of the most widely used clustering algorithms – SOM [4], K-means [15], and hierarchical clustering [3], as well as *CLICK*, a graph theoretic based algorithm developed in our lab. CLICK is described in detail in [16] and it was demonstrated to outperform other algorithms according to several figures of merit [9]. When computing a clustering solution, *EXPANDER *also specifies its homogeneity and separation measures, enabling the user to compare the merits of different solutions. Several displays for patterns (Fig. 2) and matrices (Fig. 3) are provided for the visualization of clustering solutions.

### Bicluster analysis

**Figure 4:**
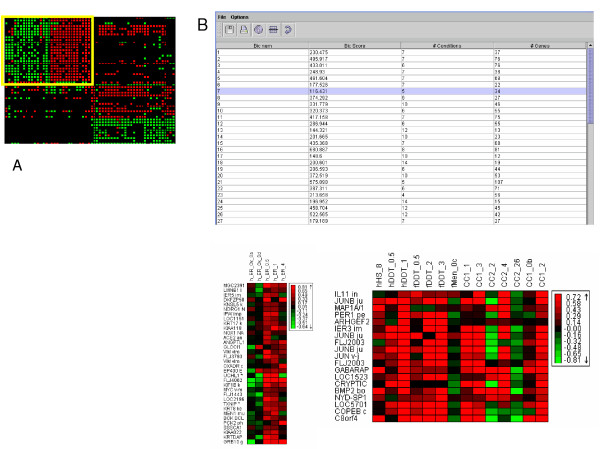
Bicluster analysis. (A) A bicluster corresponds to a submatrix defined by row and column subsets. Both subsets are not known in advance. After reordering the original data matrix, it can be seen as the rectangle with the yellow border. (B) *EXPANDER *summarizes bicluster analysis results in a table that lists the dimensions (numbers of genes and conditions) of the biclusters identified and their scores. Clicking on a row in this table pops-up a window with the submatrix view of the selected bicluster. Below the table there are two examples of biclusters identified in a dataset comprising some 1,000 genes measured across over 70 conditions in human cells. Row and column labels are gene and condition names for the bicluster, respectively.

As expression data accumulate, and profiles over hundreds of different biological conditions are readily available, clustering becomes too restrictive. Clustering algorithms globally partition genes into disjoint sets according to the overall similarity in their expression patterns, i.e., they search for genes that exhibit similar expression levels over all the measured conditions. Such requirement is appropriate when analyzing small to medium size datasets from one or a few related experiments or when analyzing time-series data, as it provides statistical robustness and produces results that are easily visualized and comprehended. Yet, when larger datasets are analyzed, a more flexible approach is frequently advantageous. A *bicluster *(or a *module*) is defined as a set of genes that exhibit significant similarity over a subset of the conditions (Fig 4a). A biclustering algorithm can dissect a large gene expression dataset into a collection of biclusters, where genes or conditions can take part in more than one bicluster. A set of biclusters can thus characterize a combined, multifaceted gene expression dataset [10]. An enhanced version of our biclustering algorithm, called *SAMBA *(**S**tatistical-**A**lgorithmic **M**ethod for **B**icluster **A**nalysis) is integrated in *EXPANDER *and is the preferable partition-analysis approach for large heterogeneous datasets that encompass dozens of conditions (Fig 4b). *SAMBA *2.0 can handle datasets with thousands of conditions profiled over entire genomes. For technical description of the SAMBA algorithm see [10,17]. Briefly, the algorithm first transforms gene expression data into a weighted bipartite graph (with genes and conditions as its two parts) and then applies a statistical scoring scheme and a combinatorial algorithm to identify heavy subgraphs in the bipartite graph. Each such heavy subgraph represents a bicluster. SAMBA operates in three phases: in the first step bicluster seeds are detected, then each seed is optimized to a locally optimal bicluster, and finally a non redundant subset of the locally optimized biclusters is selected. SAMBA 2.0 contains a new implementation of the first step in which efficient hashing techniques are now utilized, thereby significantly improving running time. It also features a new redundancy filtering algorithm (step 3) that optimizes the total likelihood of a set of biclusters using a probabilistic model that generalizes the single bicluster model.*EXPANDER *allows the user to tune *SAMBA*'s performance by selecting among several multi-level discretization schemes based on the numerical characteristics of the analyzed dataset. Another important tunable parameter controls the stringency of the redundancy-filtering algorithm.

### Functional enrichment analysis

After identifying the main co-expressed gene groups in the data (either by clustering or biclustering), one of the major challenges is to ascribe them to some biological meaning. To assist the researcher in this task, *EXPANDER *contains a statistical analysis module that seeks specific functional categories that are significantly over-represented in the analyzed gene groups, with respect to a given background set of genes. In addition to pointing to possible biological roles for distinct gene sets, such analysis was demonstrated to be very helpful in assigning putative functional roles to uncharacterized genes [10,18]. *EXPANDER *is provided with pre-compiled functional annotation files for six organisms: yeast (*S. cerevisiae*), worm (*C. Elegans*), fly (*D. melanogaster*), rat (*R. norvegicus*), mouse (*M. musculus*) and human, releasing the user from the burden of compiling such annotation information. These annotation files, compiled based on data provided by the Gene Ontology (GO) consortium [19] and the central databases for these organisms, associate genes with GO functional categories.

**Figure 5:**
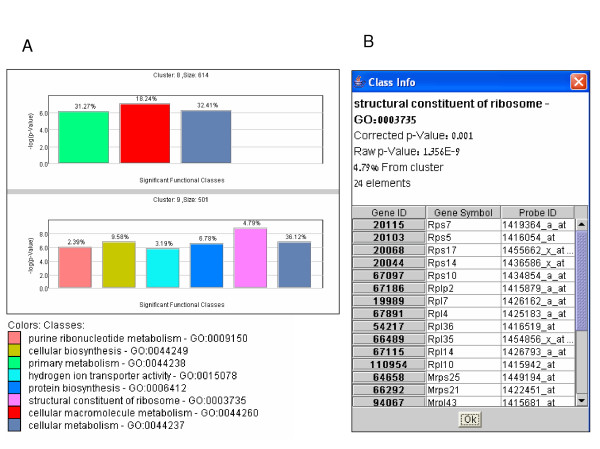
GO functional enrichment analysis. (A) Enriched GO categories identified by *TANGO *in the analyzed gene groups (clusters or biclusters) are displayed as bar diagrams; each corresponding to a specific gene group (i. e., cluster or bicluster). In these diagrams, GO categories are color-coded, and the height of a bar represents the statistical significance (-log_10_(p-value)) of the observed enrichment for its corresponding category. The percentage of genes in the group assigned to the enriched category is indicated above the bar. (B) Clicking on a bar pops-up a window that lists the group's genes that are associated with the corresponding GO category. In this window, genes are linked to central annotation DBs (SGD [25] for yeast, WormBase [26] for worm, FlyBase [27] for fly, and Entrez Gene [28] for human, mouse and rat) where detailed gene descriptions can be found for in-depth analysis.

A major challenge in identifying cases of over-represented GO categories is obtaining a good estimation of statistical significance for each case, that takes multiple testing into account (hundreds of categories are typically tested for enrichment). What complicates this task is the hierarchical tree-like structure of the ontology, which induces strong dependencies among GO categories. Thus, standard methods for accounting for multiple testing, which assume independent tests (e.g., Bonferroni, False Discovery Rate) are far too stringent. *EXPANDER *uses the *TANGO *(**T**ool for **AN**alysis of **GO **enrichments) algorithm for coping with this problem (Tanay et al., in preparation). Briefly, *TANGO *repeatedly shuffles genes to compute an empirical distribution of maximum p-values for functional enrichment obtained across a random sample of clusters that maintain the same size characteristics of the analyzed clusters. *TANGO *uses this empirical distribution to determine thresholds for significant enrichment on the true clusters. Another problem that stems from the strong dependencies among GO categories is the high level of redundancy in the reported enriched categories, which often include both parent and child nodes associated with highly overlapping set of genes. *TANGO *filters out such redundant categories by performing conditional enrichment tests that ensure that all the reported enriched categories are statistically significant even after taking into account the enrichment of their related nodes in the tree. An example for the visualization of *TANGO *results is shown in Figure 5.

### Cis-regulatory element analysis

**Figure 6:**
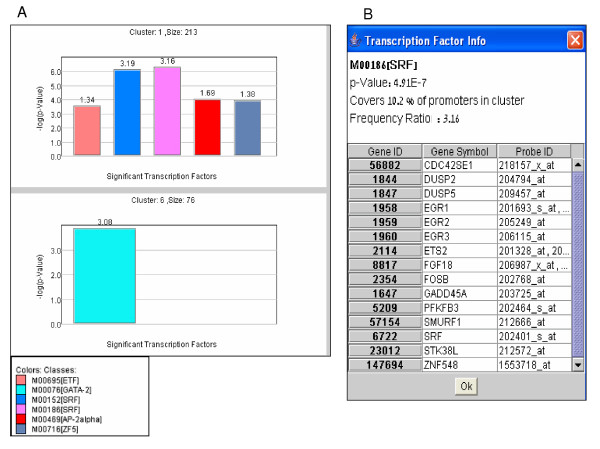
Promoter cis-regulatory elements enrichment. (A) Transcription factors (TFs) whose DNA binding site signatures are over-represented in promoters of the genes assigned to the clusters are displayed in bar diagrams. Like the display for the GO analysis (Fig. 5), each diagram corresponds to a specific gene group (cluster or bicluster), TFs are color-coded and identified by the accession number of their binding site model in TRANSFAC DB. The statistical significance of the observed enrichment for a TF is represented by the height of its bar (-log_10_(p-value)). The TF enrichment factor, which is the ratio between the prevalence of the TF hits in the gene group and in the background set of promoters, is indicated above the bar. (B) Clicking on a specific bar pops-up a window that lists the genes in the group whose promoters were found to contain a hit for that TF. In this window, genes are linked to central annotation DB of the analyzed organism as specified in the legend of Figure 5.

Microarray measurements provide snapshots of cellular transcriptional programs that take place in the examined biological conditions. These measurements do not, however, directly reveal the regulatory networks that underlie the observed transcriptional activity, i.e. the transcription factors (TFs) that control the transcription of the responding genes. Computational promoter analysis can shed light on the regulators layer of the network. Based on the assumption that genes that exhibit similar expression pattern over multiple conditions are likely to be controlled by common regulators and, therefore, share common cis-regulatory elements in their promoter regions, several algorithms have been developed to identify over-represented cis-elements in promoters of co-expressed genes. Such computational approaches successfully delineated transcriptional networks in organisms ranging from yeast to human [7,15]. *EXPANDER *provides such promoter analysis utility by integrating our *PRIMA *(**PR**omoter **I**ntegration in **M**icroarray **A**nalysis) tool which is described in detail in [7]. In short, given target sets and a background set of promoters, *PRIMA *performs statistical tests aimed at identifying transcription factors whose binding site signatures are significantly more prevalent in any of the target sets than in the background set. Typically, sets of co-expressed genes identified using either cluster or bicluster analysis serve as target sets, and the entire collection of promoters of genes present on the microarray serves as the background set. In its stand-alone version, an execution of *PRIMA *typically takes several hours to complete. To facilitate the computations of *PRIMA *from within *EXPANDER*, we added a preprocessing phase, which decreased the running time to just a few minutes on a standard PC. The preprocessing phase is run by us on occasions of major updates to genome sequence assemblies of the supported organisms (typically, once every few months). It generates promoter-fingerprints file per organism. These fingerprints files map computationally-identified high scoring putative binding sites ('*hits*') of all TFs to the entire set of promoters in the organisms. In the version integrated in *EXPANDER*, *PRIMA *loads the hits data from the fingerprints files rather than scanning promoter sequences de-novo on each run, thereby drastically reducing the running time. This improvement greatly enhanced the flexibility of *PRIMA*, enabling its execution in an iterative way, in which results obtained by different clustering solutions can be routinely compared. *EXPANDER *provides genome-wide pre-processed promoter fingerprints files for the six organisms that are we currently support (yeast, worm, fly, mouse, rat and human). The integration of *PRIMA *into *EXPANDER *allows the user to both identify the major expression patterns in his/her data (by applying *EXPANDER*'s cluster analysis module), and points to transcription factors that underlie the transcriptional alterations observed in the clusters (Fig 6).

### Demonstration of *EXPANDER*'s capabilities

To demonstrate the utility of the *EXPANDER *package, we applied it to a very large dataset published recently by Murray et al [20]. This study recorded expression profiles in several human cell lines exposed to various stressful conditions. The authors integrated these data with a dataset in which expression profiles were measured throughout the progression of the cell cycle [21]. The combined dataset contains expression data for 36,825 probes measured over 174 conditions. The analysis of such complex dataset poses a daunting bioinformatics challenge. Murray et al. used the Cluster/TreeView tool [3] to hierarchically cluster this dataset, and by visual inspection of the resulting tree defined the main clusters in the data. A second "adoption step" was then applied, in which each main cluster adopted genes whose expression pattern resembled the cluster's mean pattern. Overall, 23 clusters containing 1245 distinct genes were reported. Biological meaning was assigned to the clusters by inspection of their expression profiles and of the genes they contain. No promoter analysis was reported.

As we noted above, when analyzing large datasets, biclustering becomes more appropriate than clustering. Therefore, we subjected this dataset to bicluster analysis using SAMBA. We first replaced missing entries with 0 (which corresponds to 'no change' in log-transformed data) and then scanned the dataset for probes whose expression was changed by at least 2-fold in at least 7 conditions. Some 10% of the clones (3,392) passed this filtering. We applied SAMBA to the union of these genes and the 1245 genes analyzed by Murray et al. The union contains 3892 genes. SAMBA identified 155 biclusters on this filtered dataset. (These biclusters can overlap – genes can be assigned to several biclusters – but are not redundant: a pruning step removes highly overlapping biclusters.) The identified biclusters reveal the major expression patterns that underlie this intricate dataset. Next, we aimed to assign biclusters with putative functional meaning, and to identify major TFs that regulate the transcriptional responses captured by them. To this goal, we applied the TANGO and PRIMA modules (both were run with default parameters).

The purpose of this exercise is not to apply in-depth biological analysis of stress responses in human cells, but to demonstrate the strength and agility of *EXPANDER *in analysis of complex microarray datasets. Therefore we only briefly summarize some of the major biclusters identified in the dataset, along with their putative biological roles and transcriptional regulators that were computationally discovered by *EXPANDER*. The major biclusters identified are listed in Table 1 and some of them are presented in Additional file 1. In agreement with Murray et al., we found that most of the transcriptional responses to stressful conditions were agent- and cell-type- specific (for example, bicluster #1 represents 145 genes that were activated only in Hela cells exposed to heat shock; bicluster #24 represents over 100 genes that were activated only in fibroblasts exposed to DDT). In addition, some biclusters correspond to more general stress responses that were induced by multiple agents and in different cell lines (for example, bicluster #53 contains 51 genes that were down-regulated in response to both oxidative stress and heat shock in Hela cells and in response to heat shock in fibroblasts). As pointed by Murray et al, analyzing together the stress data and the cell-cycle data allows the distinction between genes that respond directly to the stress agents and those whose change can be explained by differences in the fraction of cells in the different phases of the cell cycle due to activation of cell cycle checkpoints after exposure to damaging agents. Indeed, bicluster #106 is enriched for DNA replication genes, up-regulated in S-phase time points in the cell-cycle dataset, and down-regulated in fibroblasts exposed to either DDT or Menadione, probably reflecting an arrest of these cells in early G1 or G2 phase. Similarly, biclusters #40 and #104 show the down-regulation of mitotic genes in several cell lines and in response to various stress agents, probably reflecting the reduction in the fraction of cells undergoing mitosis in these stressed cell populations.

**Table 1:** Major biclusters identified in the test case analysis of the human stress data set.

**Bicluster number**	**Num of Conditions**	**Num of Genes**	**Enriched GO (GOid, p-val)**	**Enriched TF binding site signatures (TRANSFAC id, p-val)**	**Comments**
106	9	79	DNA Replication (GO:0006260, 5.3 × 10^-9^)	E2F (M00918, 1.3 × 10^-7^)	Down-regulation of DNA replication genes in fibroblasts exposed to DDT or Menadione.
40	33	74	Mitosis (GO:0007067, 9.3 × 10^-19^)	NF-Y (M00287, 6.7 × 10^-22^) IRF-7 (M00453, 9.5 × 10^-5^)	Down-regulation of mitotic genes in response to various stresses.
104	41	13	Mitosis (GO:0007067, 3.3 × 10^-10^)	NF-Y (M00287, 3.4 × 10^-9^)	Down-regulation of mitotic genes in response to various stresses.
16	5	89	Carboxylic acid metabolism (GO:0019752, 3.4 × 10^-8^)	---	Genes activated in Hela cells in response to Tunicamycin and Menadione
1	6	145	Response to unfolded protein (GO:0006986, 1.2 × 10^-7^)	---	Genes activated in Hela cells in response to heat shock
9	7	142	Response to unfolded protein (GO:0006986, 7.3 × 10^-9^)	AP-2alpha (M00469, 5.6 × 10^-4^)	Genes activated in K562 cells in response to heat shock
111	10	24	Response to unfolded protein (GO:0006986, 1.5 × 10^-7^)	---	Genes that are activated by heat shock but repressed by crowding in Hela cells
24	6	105	Transcription corepressor (GO:0003714, 1.5 × 10^-6^)	HIF-1 (M00797, 6.9 × 10^-4^)	Genes activated in fibroblasts in response to DDT
27	8	200	---	----	Genes activated in fibroblasts in response to oxidative stress (H2O2)
61	9	134	---	----	Genes that are repressed by crowding in fibroblasts.
53	22	51	----	N-Myc(M00055, 2.7 × 10^-6^)	Genes that are repressed in both Hela cells and fibroblasts.
89	9	115	---	AP-4 (M00005, 2.1 × 10^-4^)	Genes repressed in Hela cells in response to various stresses.
123	10	31	---	NFkB (M00051, 7.1 × 10^-4^)	Genes activated in Hela cells in response to DDT.

In several biclusters PRIMA identified significant enrichment for binding site signatures of TFs that are known to control the respective biological processes (e.g., over-representation of E2F binding site in bicluster #106, which is enriched for DNA replication genes; enrichment of NF-Y binding site in bicluster #40, which is enriched for mitotic genes). In other biclusters PRIMA suggests novel links between TFs and stress responses (e.g., over-representation of N-Myc binding site in bicluster #53, which contains genes that are repressed by different stresses).

Some of *EXPANDER*'s salient advantages are evident from the above analysis: The biclustering module, which is unique to *EXPANDER *among packages for microarray data analysis, allows systematic detection of the major expression patterns in highly complex datasets. Biclusters provide higher resolution gene groups, some encompassing many conditions but most covering relatively small subsets and thus focusing on specific phenomena. Functional enrichment and promoter analyses are done in a streamlined and integrated fashion, and so most of the expert's effort can be devoted to biological interpretation. Last, analysis of microarray data requires experimentation with various filtering thresholds and algorithmic parameters settings; therefore it is of high importance that the analysis modules will require relatively short running time. *EXPANDER *was designed to meet this requirement. A full analysis iteration, which includes biclustering, functional enrichment and promoter analyses applied to the above massive dataset that we used as an example, takes some 15 mins on a standard PC.

#### Comparison with other tools

Several integrative packages for the analysis of gene expression data were are available, among them are INCLUSive [22], Expression-Profiler, GEPAS [23], TIGR's Multiple Experiment Viewer, and ArrayPipe [24]. *EXPANDER *has several advantages over extant packages. While some of the integrative packages are designed as web portals that provide links to independent programs, where, in some cases, the outputs are sent to the user by e-mail and not always in a format directly compatible with subsequent analysis steps, in *EXPANDER *the analysis flow is inherently streamlined and straightforward. In addition, *EXPANDERs*' strength lies in the advanced algorithms it uniquely provides: CLICK for clustering, SAMBA for biclustering, TANGO for identification of GO enrichment, and PRIMA for the identification of enriched TF binding site signatures. The synergism that stems from the integration of these algorithms into one package grants *EXPANDER *with very powerful analytical capabilities. Another feature that distinguishes *EXPANDER *is its built-in support for genome-wide analysis of data obtained from six major research organisms.

## Conclusion

Designed as a 'one-stop shop' for gene expression data analysis, *EXPANDER *provides algorithms covering main analysis steps including (1) the initial process of normalization and filtering for removing biases and focusing downstream analysis on responding genes in the dataset; (2) clustering and biclustering to discover the main expression patterns in the data; (3) high-level functional enrichment analysis; and (4) promoter cis-element analysis to gain insights on the biological meaning of the identified expression patterns and to point to transcriptional regulators that underlie them. These integrated capabilities provided by *EXPANDER *and its built-in support of multiple organisms make it a very powerful tool for analysis of microarray data. Although some of the analysis modules implemented in *EXPANDER *are based on sophisticated algorithms, their execution remains simple and intuitive.

We will routinely post on *EXPANDER'*s website updated GO annotation and promoter fingerprint files for all the supported organisms. *EXPANDER*'s users will be notified of such updates. We will continue to maintain and expand *EXPANDER *to keep it as an integrative suite that provides state-of-the-art algorithms and visualization utilities for analysis of microarray data. We will also expand the group of organisms supported by the package according to the availability of appropriate information and data.

## Availability and requirements

• Project name: EXPANDER

• Project home page: 

• Operating system(s): Windows, UNIX

• Programming language: Java for the envelope and C for most of the algorithms.

• Other requirements: Java 1.4 or higher

• License: free for non-commercial users.

• Any restrictions to use by non-academics: License needed.

## Abbreviations

EXPANDER – EXPression ANalyzer and DisplayER

SOM – Self Organizing Maps

CLICK – CLuster Identification via Connectivity Kernels

SAMBA – Statistical-Algorithmic Method for Bicluster Analysis

TANGO – Tool for ANalysis of GO enrichments

PRIMA – PRomoter Integration in Microarray Analysis

GO – Gene Ontology

PWM – Position weight matrix

TF – Transcription factor

## Authors' contributions

R. Sharan developed CLICK. AT and R. Sharan developed SAMBA, and AT with IS improved and enhanced it. CL and RE developed PRIMA. AT developed TANGO. AM coded and integrated the complete package and the visualization methods. RE and CL created the annotation and promoter files for the six species. RE performed the use-case data analysis and wrote the manuscript with R. Shamir. R. Shamir, with help from YS and RE, conceived, designed and led the project.

## Supplementary Material

Additional File 1 Examples of 13 major biclusters identified on the human cells stress-response dataset. Enriched GO categories and TFBS signature found in these biclusters are summarized in Table 1. In each matrix, rows and columns correspond, respectively, to genes and conditions that participate in the bicluster. Labels of the conditions follow this convention: the cell line is indicated first (Hela for Hela cells, WI for WI38 fibroblasts, or K for K-562 cells), followed by an indication for the stress agent (HS – heat shock, DDT, Ox – H2O2 oxidative stress, Crd – crowding, Men – Menadione, Tun – Tunicamycin, CC – cell cycle data measured in Hela cells synchronized using double thymidine, and CCb – cell cycle data in Hela cells synchronized using thymidine-nocodazole). The last number in the label indicates the time point. Click here for file

## References

[B1] Bolstad BM, Irizarry RA, Astrand M, Speed TP (2003). A comparison of normalization methods for high density oligonucleotide array data based on variance and bias. Bioinformatics.

[B2] Irizarry RA, Hobbs B, Collin F, Beazer-Barclay YD, Antonellis KJ, Scherf U, Speed TP (2003). Exploration, normalization, and summaries of high density oligonucleotide array probe level data. Biostatistics.

[B3] Eisen MB, Spellman PT, Brown PO, Botstein D (1998). Cluster analysis and display of genome-wide expression patterns. Proc Natl Acad Sci U S A.

[B4] Tamayo P, Slonim D, Mesirov J, Zhu Q, Kitareewan S, Dmitrovsky E, Lander ES, Golub TR (1999). Interpreting patterns of gene expression with self-organizing maps: methods and application to hematopoietic differentiation. Proc Natl Acad Sci U S A.

[B5] Al-Shahrour F, Diaz-Uriarte R, Dopazo J (2004). FatiGO: a web tool for finding significant associations of Gene Ontology terms with groups of genes. Bioinformatics.

[B6] Aerts S, Thijs G, Coessens B, Staes M, Moreau Y, De Moor B (2003). Toucan: deciphering the cis-regulatory logic of coregulated genes. Nucleic Acids Res.

[B7] Elkon R, Linhart C, Sharan R, Shamir R, Shiloh Y (2003). Genome-wide in silico identification of transcriptional regulators controlling the cell cycle in human cells. Genome Res.

[B8] Sharan R, Maron-Katz A, Shamir R (2003). CLICK and EXPANDER: a system for clustering and visualizing gene expression data. Bioinformatics.

[B9] Sharan R, Elkon R, Shamir R (2002). Cluster analysis and its applications to gene expression data. Ernst Schering Res Found Workshop.

[B10] Tanay A, Sharan R, Kupiec M, Shamir R (2004). Revealing modularity and organization in the yeast molecular network by integrated analysis of highly heterogeneous genomewide data. Proc Natl Acad Sci U S A.

[B11] Schuchhardt J, Beule D, Malik A, Wolski E, Eickhoff H, Lehrach H, Herzel H (2000). Normalization strategies for cDNA microarrays. Nucleic Acids Res.

[B12] TP. S, Yang YH, Dudoit S, Luu P (2000). Normalization for cDNA Microarray Data. Technical Report Department of Statistics, University of California at Berkeley.

[B13] Huber W, von Heydebreck A, Sultmann H, Poustka A, Vingron M (2002). Variance stabilization applied to microarray data calibration and to the quantification of differential expression. Bioinformatics.

[B14] Kel AE, Kel-Margoulis OV, Farnham PJ, Bartley SM, Wingender E, Zhang MQ (2001). Computer-assisted identification of cell cycle-related genes: new targets for E2F transcription factors. J Mol Biol.

[B15] Tavazoie S, Hughes JD, Campbell MJ, Cho RJ, Church GM (1999). Systematic determination of genetic network architecture. Nat Genet.

[B16] Sharan R, Shamir R (2000). CLICK: a clustering algorithm with applications to gene expression analysis. Proc Int Conf Intell Syst Mol Biol.

[B17] Tanay A, Sharan R, Shamir R (2002). Discovering statistically significant biclusters in gene expression data. Bioinformatics.

[B18] Wu LF, Hughes TR, Davierwala AP, Robinson MD, Stoughton R, Altschuler SJ (2002). Large-scale prediction of Saccharomyces cerevisiae gene function using overlapping transcriptional clusters. Nat Genet.

[B19] Ashburner M, Ball CA, Blake JA, Botstein D, Butler H, Cherry JM, Davis AP, Dolinski K, Dwight SS, Eppig JT, Harris MA, Hill DP, Issel-Tarver L, Kasarskis A, Lewis S, Matese JC, Richardson JE, Ringwald M, Rubin GM, Sherlock G (2000). Gene ontology: tool for the unification of biology. The Gene Ontology Consortium. Nat Genet.

[B20] Murray JI, Whitfield ML, Trinklein ND, Myers RM, Brown PO, Botstein D (2004). Diverse and specific gene expression responses to stresses in cultured human cells. Mol Biol Cell.

[B21] Whitfield ML, Sherlock G, Saldanha AJ, Murray JI, Ball CA, Alexander KE, Matese JC, Perou CM, Hurt MM, Brown PO, Botstein D (2002). Identification of genes periodically expressed in the human cell cycle and their expression in tumors. Mol Biol Cell.

[B22] Coessens B, Thijs G, Aerts S, Marchal K, De Smet F, Engelen K, Glenisson P, Moreau Y, Mathys J, De Moor B (2003). INCLUSive: A web portal and service registry for microarray and regulatory sequence analysis. Nucleic Acids Res.

[B23] Herrero J, Vaquerizas JM, Al-Shahrour F, Conde L, Mateos A, Diaz-Uriarte JS, Dopazo J (2004). New challenges in gene expression data analysis and the extended GEPAS. Nucleic Acids Res.

[B24] Hokamp K, Roche FM, Acab M, Rousseau ME, Kuo B, Goode D, Aeschliman D, Bryan J, Babiuk LA, Hancock RE, Brinkman FS (2004). ArrayPipe: a flexible processing pipeline for microarray data. Nucleic Acids Res.

[B25] Christie KR, Weng S, Balakrishnan R, Costanzo MC, Dolinski K, Dwight SS, Engel SR, Feierbach B, Fisk DG, Hirschman JE, Hong EL, Issel-Tarver L, Nash R, Sethuraman A, Starr B, Theesfeld CL, Andrada R, Binkley G, Dong Q, Lane C, Schroeder M, Botstein D, Cherry JM (2004). Saccharomyces Genome Database (SGD) provides tools to identify and analyze sequences from Saccharomyces cerevisiae and related sequences from other organisms. Nucleic Acids Res.

[B26] Chen N, Harris TW, Antoshechkin I, Bastiani C, Bieri T, Blasiar D, Bradnam K, Canaran P, Chan J, Chen CK, Chen WJ, Cunningham F, Davis P, Kenny E, Kishore R, Lawson D, Lee R, Muller HM, Nakamura C, Pai S, Ozersky P, Petcherski A, Rogers A, Sabo A, Schwarz EM, Van Auken K, Wang Q, Durbin R, Spieth J, Sternberg PW, Stein LD (2005). WormBase: a comprehensive data resource for Caenorhabditis biology and genomics. Nucleic Acids Res.

[B27] Drysdale RA, Crosby MA, Gelbart W, Campbell K, Emmert D, Matthews B, Russo S, Schroeder A, Smutniak F, Zhang P, Zhou P, Zytkovicz M, Ashburner M, de Grey A, Foulger R, Millburn G, Sutherland D, Yamada C, Kaufman T, Matthews K, DeAngelo A, Cook RK, Gilbert D, Goodman J, Grumbling G, Sheth H, Strelets V, Rubin G, Gibson M, Harris N, Lewis S, Misra S, Shu SQ (2005). FlyBase: genes and gene models. Nucleic Acids Res.

[B28] Maglott D, Ostell J, Pruitt KD, Tatusova T (2005). Entrez Gene: gene-centered information at NCBI. Nucleic Acids Res.

