# Supplementary figures and images for: EXPANDER – an integrative program suite for microarray data analysis
Source: BMC Bioinformatics. 2005 Sep 21;6:232. doi: 10.1186/1471-2105-6-232 (PMC1261157; doi:10.1186/1471-2105-6-232)

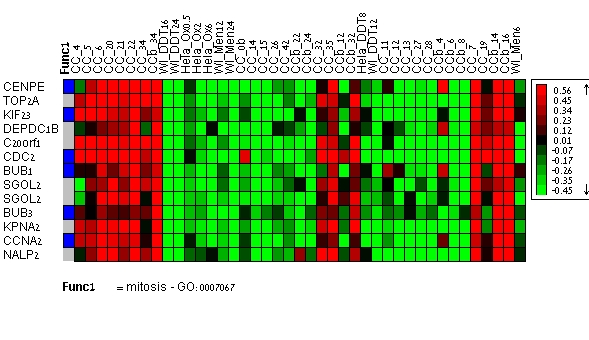

Supplement: Additional File 1 — Examples of 13 major biclusters identified on the human cells stress-response dataset. Enriched GO categories and TFBS signature found in these biclusters are summarized in Table 1. In each matrix, rows and columns correspond, respectively, to genes and conditions that participate in the bicluster. Labels of the conditions follow this convention: the cell line is indicated first (Hela for Hela cells, WI for WI38 fibroblasts, or K for K-562 cells), followed by an indication for the stress agent (HS – heat shock, DDT, Ox – H2O2 oxidative stress, Crd – crowding, Men – Menadione, Tun – Tunicamycin, CC – cell cycle data measured in Hela cells synchronized using double thymidine, and CCb – cell cycle data in Hela cells synchronized using thymidine-nocodazole). The last number in the label indicates the time point. [file 1471-2105-6-232-S1.zip › Supp_Fig_bic_104_Mitosis.jpg]

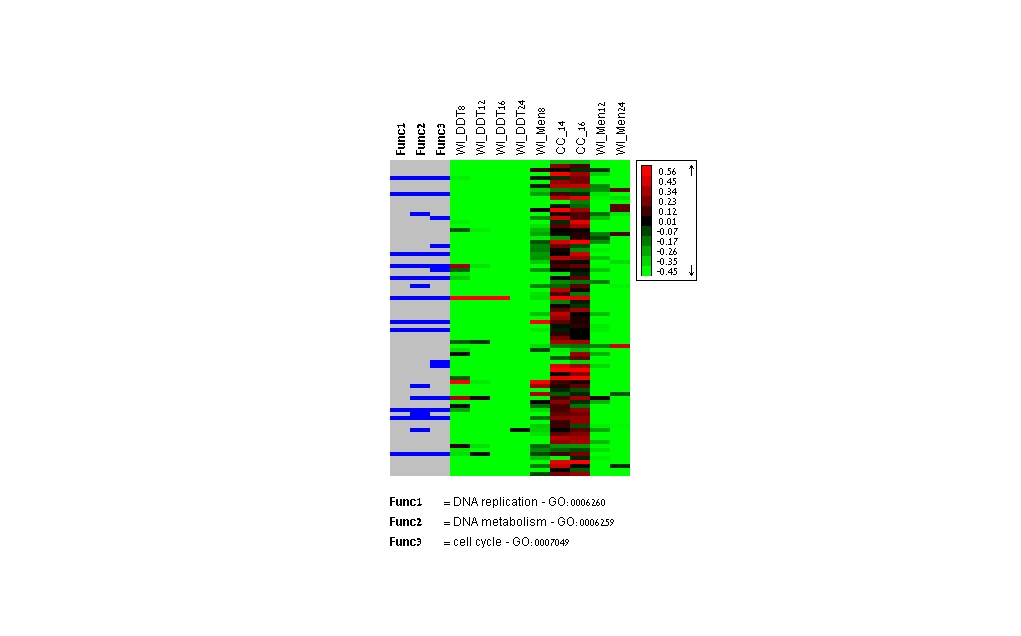

Supplement: Additional File 1 — Examples of 13 major biclusters identified on the human cells stress-response dataset. Enriched GO categories and TFBS signature found in these biclusters are summarized in Table 1. In each matrix, rows and columns correspond, respectively, to genes and conditions that participate in the bicluster. Labels of the conditions follow this convention: the cell line is indicated first (Hela for Hela cells, WI for WI38 fibroblasts, or K for K-562 cells), followed by an indication for the stress agent (HS – heat shock, DDT, Ox – H2O2 oxidative stress, Crd – crowding, Men – Menadione, Tun – Tunicamycin, CC – cell cycle data measured in Hela cells synchronized using double thymidine, and CCb – cell cycle data in Hela cells synchronized using thymidine-nocodazole). The last number in the label indicates the time point. [file 1471-2105-6-232-S1.zip › Supp_Fig_bic_106_DNA_Rep.jpg]

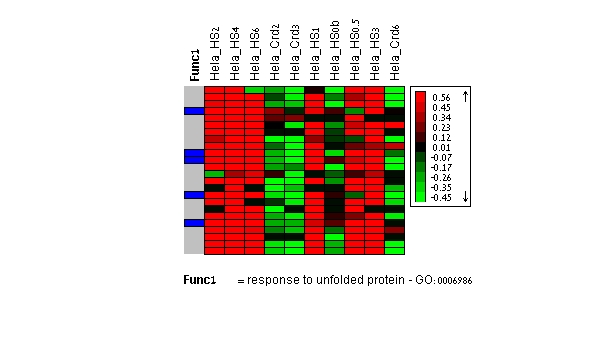

Supplement: Additional File 1 — Examples of 13 major biclusters identified on the human cells stress-response dataset. Enriched GO categories and TFBS signature found in these biclusters are summarized in Table 1. In each matrix, rows and columns correspond, respectively, to genes and conditions that participate in the bicluster. Labels of the conditions follow this convention: the cell line is indicated first (Hela for Hela cells, WI for WI38 fibroblasts, or K for K-562 cells), followed by an indication for the stress agent (HS – heat shock, DDT, Ox – H2O2 oxidative stress, Crd – crowding, Men – Menadione, Tun – Tunicamycin, CC – cell cycle data measured in Hela cells synchronized using double thymidine, and CCb – cell cycle data in Hela cells synchronized using thymidine-nocodazole). The last number in the label indicates the time point. [file 1471-2105-6-232-S1.zip › Supp_Fig_bic_111_Hela_HS_Crd.jpg]

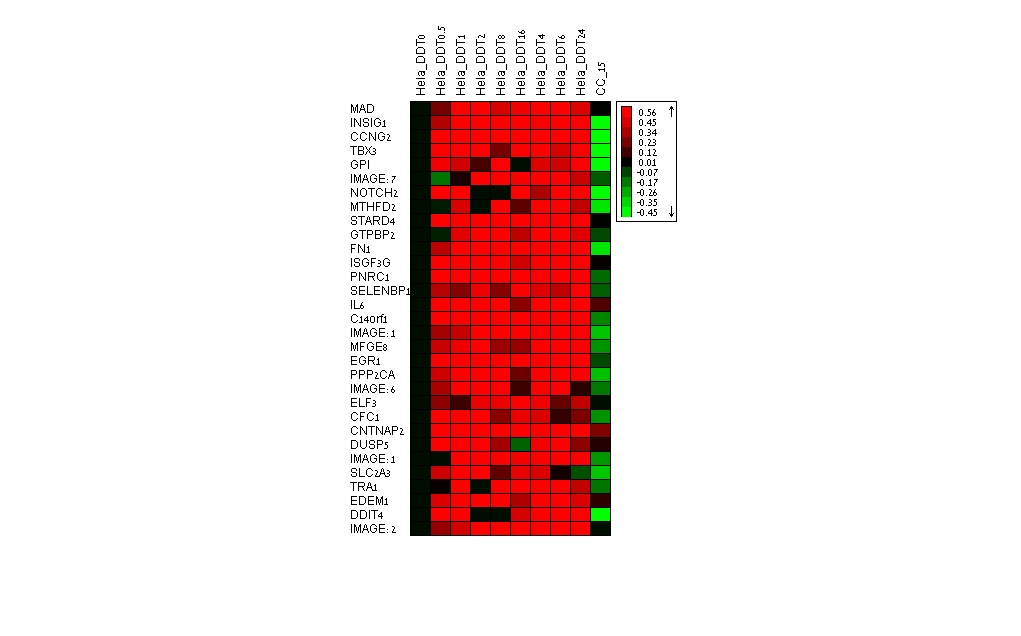

Supplement: Additional File 1 — Examples of 13 major biclusters identified on the human cells stress-response dataset. Enriched GO categories and TFBS signature found in these biclusters are summarized in Table 1. In each matrix, rows and columns correspond, respectively, to genes and conditions that participate in the bicluster. Labels of the conditions follow this convention: the cell line is indicated first (Hela for Hela cells, WI for WI38 fibroblasts, or K for K-562 cells), followed by an indication for the stress agent (HS – heat shock, DDT, Ox – H2O2 oxidative stress, Crd – crowding, Men – Menadione, Tun – Tunicamycin, CC – cell cycle data measured in Hela cells synchronized using double thymidine, and CCb – cell cycle data in Hela cells synchronized using thymidine-nocodazole). The last number in the label indicates the time point. [file 1471-2105-6-232-S1.zip › Supp_Fig_bic_123_DDT.jpg]

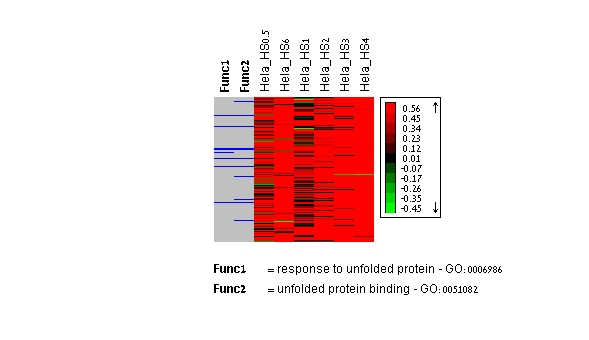

Supplement: Additional File 1 — Examples of 13 major biclusters identified on the human cells stress-response dataset. Enriched GO categories and TFBS signature found in these biclusters are summarized in Table 1. In each matrix, rows and columns correspond, respectively, to genes and conditions that participate in the bicluster. Labels of the conditions follow this convention: the cell line is indicated first (Hela for Hela cells, WI for WI38 fibroblasts, or K for K-562 cells), followed by an indication for the stress agent (HS – heat shock, DDT, Ox – H2O2 oxidative stress, Crd – crowding, Men – Menadione, Tun – Tunicamycin, CC – cell cycle data measured in Hela cells synchronized using double thymidine, and CCb – cell cycle data in Hela cells synchronized using thymidine-nocodazole). The last number in the label indicates the time point. [file 1471-2105-6-232-S1.zip › Supp_Fig_bic_1_HS_Hela.jpg]

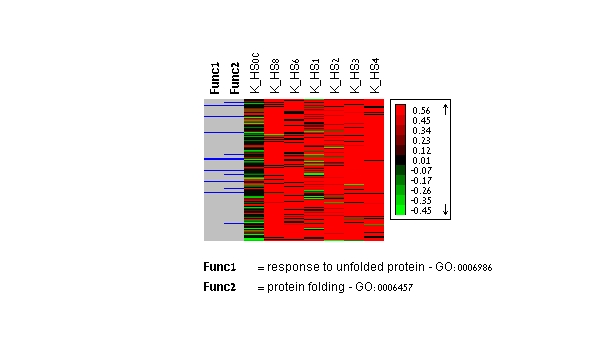

Supplement: Additional File 1 — Examples of 13 major biclusters identified on the human cells stress-response dataset. Enriched GO categories and TFBS signature found in these biclusters are summarized in Table 1. In each matrix, rows and columns correspond, respectively, to genes and conditions that participate in the bicluster. Labels of the conditions follow this convention: the cell line is indicated first (Hela for Hela cells, WI for WI38 fibroblasts, or K for K-562 cells), followed by an indication for the stress agent (HS – heat shock, DDT, Ox – H2O2 oxidative stress, Crd – crowding, Men – Menadione, Tun – Tunicamycin, CC – cell cycle data measured in Hela cells synchronized using double thymidine, and CCb – cell cycle data in Hela cells synchronized using thymidine-nocodazole). The last number in the label indicates the time point. [file 1471-2105-6-232-S1.zip › Supp_Fig_bic_9_HS_K.jpg]

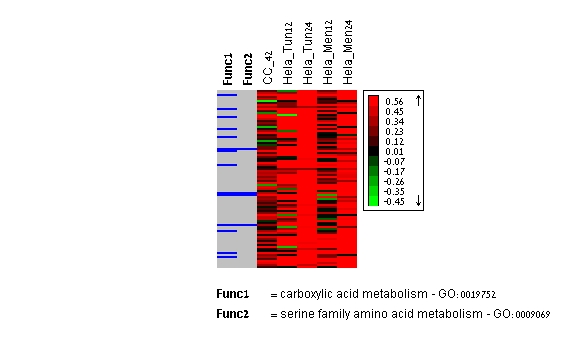

Supplement: Additional File 1 — Examples of 13 major biclusters identified on the human cells stress-response dataset. Enriched GO categories and TFBS signature found in these biclusters are summarized in Table 1. In each matrix, rows and columns correspond, respectively, to genes and conditions that participate in the bicluster. Labels of the conditions follow this convention: the cell line is indicated first (Hela for Hela cells, WI for WI38 fibroblasts, or K for K-562 cells), followed by an indication for the stress agent (HS – heat shock, DDT, Ox – H2O2 oxidative stress, Crd – crowding, Men – Menadione, Tun – Tunicamycin, CC – cell cycle data measured in Hela cells synchronized using double thymidine, and CCb – cell cycle data in Hela cells synchronized using thymidine-nocodazole). The last number in the label indicates the time point. [file 1471-2105-6-232-S1.zip › Supp_Fig_bic_16_carboxyMetabolism.jpg]

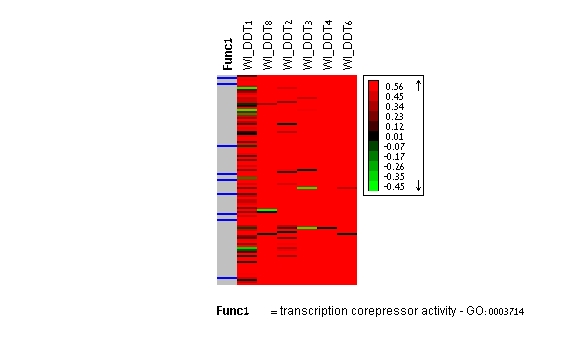

Supplement: Additional File 1 — Examples of 13 major biclusters identified on the human cells stress-response dataset. Enriched GO categories and TFBS signature found in these biclusters are summarized in Table 1. In each matrix, rows and columns correspond, respectively, to genes and conditions that participate in the bicluster. Labels of the conditions follow this convention: the cell line is indicated first (Hela for Hela cells, WI for WI38 fibroblasts, or K for K-562 cells), followed by an indication for the stress agent (HS – heat shock, DDT, Ox – H2O2 oxidative stress, Crd – crowding, Men – Menadione, Tun – Tunicamycin, CC – cell cycle data measured in Hela cells synchronized using double thymidine, and CCb – cell cycle data in Hela cells synchronized using thymidine-nocodazole). The last number in the label indicates the time point. [file 1471-2105-6-232-S1.zip › Supp_Fig_bic_24_DDT.jpg]

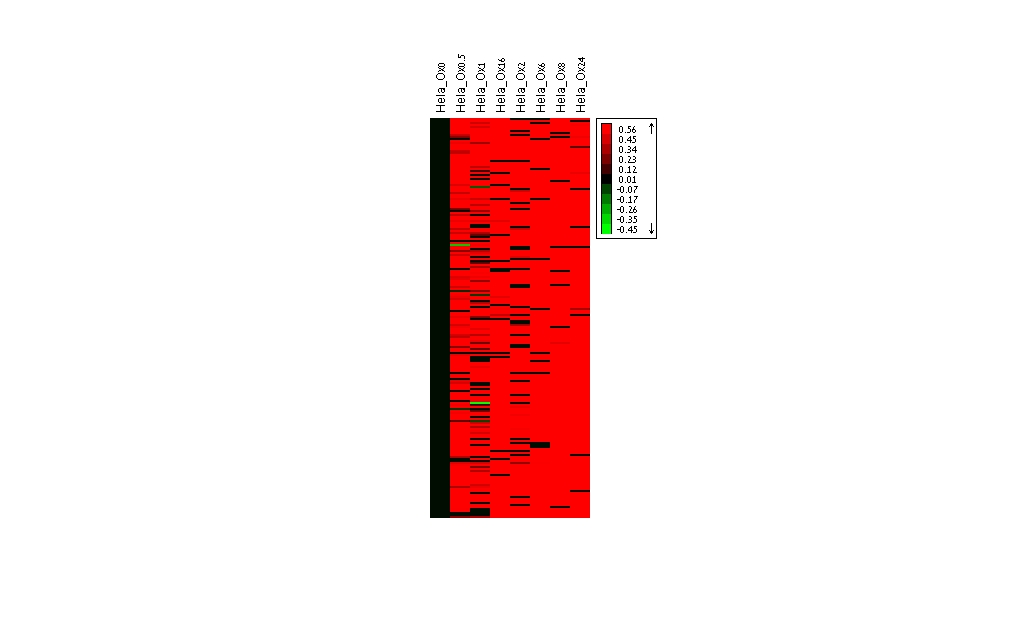

Supplement: Additional File 1 — Examples of 13 major biclusters identified on the human cells stress-response dataset. Enriched GO categories and TFBS signature found in these biclusters are summarized in Table 1. In each matrix, rows and columns correspond, respectively, to genes and conditions that participate in the bicluster. Labels of the conditions follow this convention: the cell line is indicated first (Hela for Hela cells, WI for WI38 fibroblasts, or K for K-562 cells), followed by an indication for the stress agent (HS – heat shock, DDT, Ox – H2O2 oxidative stress, Crd – crowding, Men – Menadione, Tun – Tunicamycin, CC – cell cycle data measured in Hela cells synchronized using double thymidine, and CCb – cell cycle data in Hela cells synchronized using thymidine-nocodazole). The last number in the label indicates the time point. [file 1471-2105-6-232-S1.zip › Supp_Fig_bic_27_Ox.jpg]

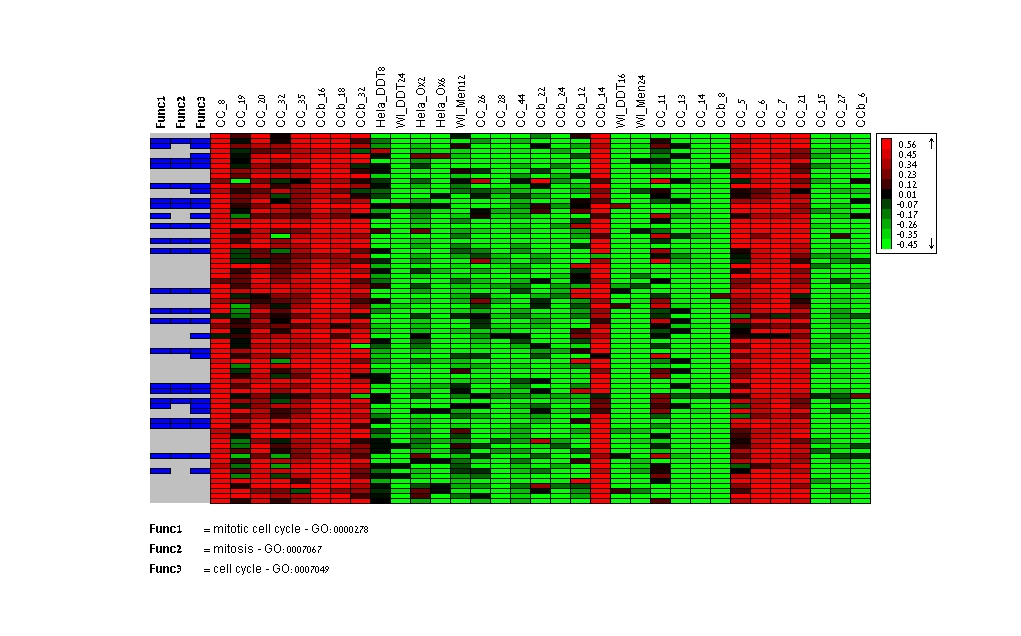

Supplement: Additional File 1 — Examples of 13 major biclusters identified on the human cells stress-response dataset. Enriched GO categories and TFBS signature found in these biclusters are summarized in Table 1. In each matrix, rows and columns correspond, respectively, to genes and conditions that participate in the bicluster. Labels of the conditions follow this convention: the cell line is indicated first (Hela for Hela cells, WI for WI38 fibroblasts, or K for K-562 cells), followed by an indication for the stress agent (HS – heat shock, DDT, Ox – H2O2 oxidative stress, Crd – crowding, Men – Menadione, Tun – Tunicamycin, CC – cell cycle data measured in Hela cells synchronized using double thymidine, and CCb – cell cycle data in Hela cells synchronized using thymidine-nocodazole). The last number in the label indicates the time point. [file 1471-2105-6-232-S1.zip › Supp_Fig_bic_40_Mitosis.jpg]

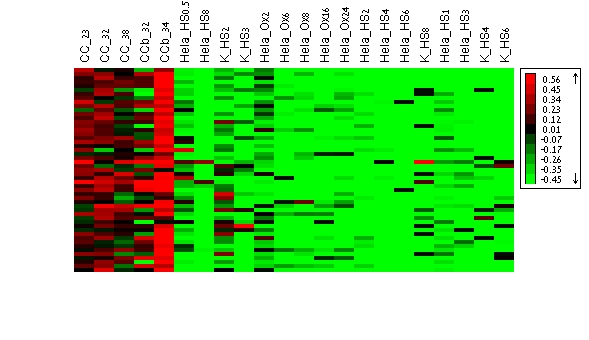

Supplement: Additional File 1 — Examples of 13 major biclusters identified on the human cells stress-response dataset. Enriched GO categories and TFBS signature found in these biclusters are summarized in Table 1. In each matrix, rows and columns correspond, respectively, to genes and conditions that participate in the bicluster. Labels of the conditions follow this convention: the cell line is indicated first (Hela for Hela cells, WI for WI38 fibroblasts, or K for K-562 cells), followed by an indication for the stress agent (HS – heat shock, DDT, Ox – H2O2 oxidative stress, Crd – crowding, Men – Menadione, Tun – Tunicamycin, CC – cell cycle data measured in Hela cells synchronized using double thymidine, and CCb – cell cycle data in Hela cells synchronized using thymidine-nocodazole). The last number in the label indicates the time point. [file 1471-2105-6-232-S1.zip › Supp_Fig_bic_53_GeneralDown.jpg]

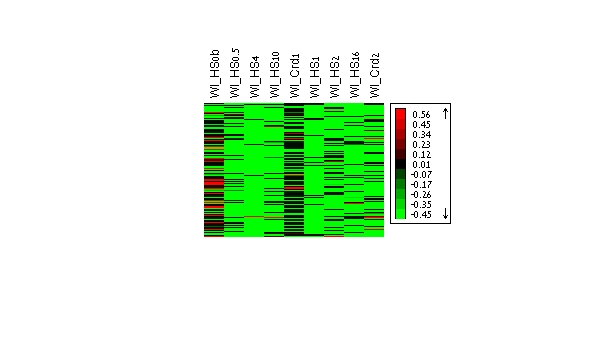

Supplement: Additional File 1 — Examples of 13 major biclusters identified on the human cells stress-response dataset. Enriched GO categories and TFBS signature found in these biclusters are summarized in Table 1. In each matrix, rows and columns correspond, respectively, to genes and conditions that participate in the bicluster. Labels of the conditions follow this convention: the cell line is indicated first (Hela for Hela cells, WI for WI38 fibroblasts, or K for K-562 cells), followed by an indication for the stress agent (HS – heat shock, DDT, Ox – H2O2 oxidative stress, Crd – crowding, Men – Menadione, Tun – Tunicamycin, CC – cell cycle data measured in Hela cells synchronized using double thymidine, and CCb – cell cycle data in Hela cells synchronized using thymidine-nocodazole). The last number in the label indicates the time point. [file 1471-2105-6-232-S1.zip › Supp_Fig_bic_61_HSandCrd_down.jpg]

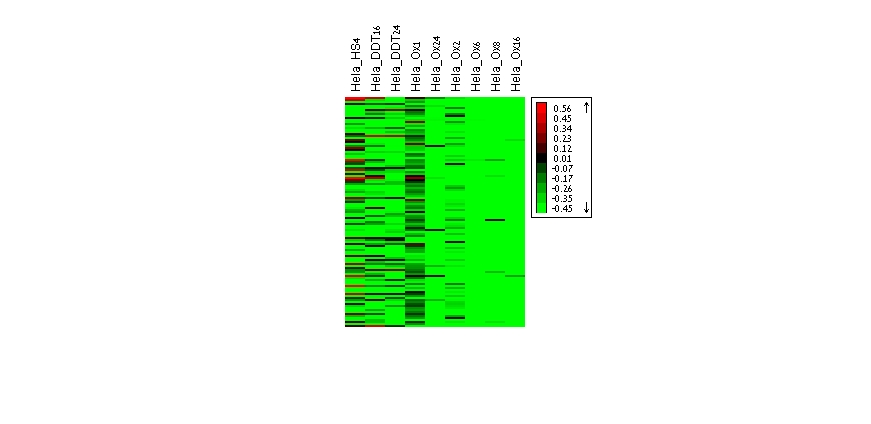

Supplement: Additional File 1 — Examples of 13 major biclusters identified on the human cells stress-response dataset. Enriched GO categories and TFBS signature found in these biclusters are summarized in Table 1. In each matrix, rows and columns correspond, respectively, to genes and conditions that participate in the bicluster. Labels of the conditions follow this convention: the cell line is indicated first (Hela for Hela cells, WI for WI38 fibroblasts, or K for K-562 cells), followed by an indication for the stress agent (HS – heat shock, DDT, Ox – H2O2 oxidative stress, Crd – crowding, Men – Menadione, Tun – Tunicamycin, CC – cell cycle data measured in Hela cells synchronized using double thymidine, and CCb – cell cycle data in Hela cells synchronized using thymidine-nocodazole). The last number in the label indicates the time point. [file 1471-2105-6-232-S1.zip › Supp_Fig_bic_89_GeneralDown.jpg]
